# Adiponectin attenuates profibrotic extracellular matrix remodeling following cardiac injury by up‐regulating matrix metalloproteinase 9 expression in mice

**DOI:** 10.14814/phy2.13523

**Published:** 2017-12-21

**Authors:** Alexander Jenke, Robert Schur, Carsten Röger, Zehra Karadeniz, Mathias Grüger, Luise Holzhauser, Kostas Savvatis, Wolfgang Poller, Heinz‐Peter Schultheiss, Ulf Landmesser, Carsten Skurk

**Affiliations:** ^1^ Department of Cardiology Charité University Medicine Berlin Campus Benjamin Franklin Berlin Germany; ^2^ Department of Internal Medicine Albert‐Einstein College of Medicine Bronx New York; ^3^ Department of Cardiology Barts Heart Centre Barts Health NHS Trust London United Kingdom; ^4^ DZHK (German Centre for Cardiovascular Research) partner site Berlin Germany; ^5^ Berlin Institute of Health (BIH) Berlin Germany

**Keywords:** Adiponectin, ECM remodeling, fibrosis, MMP‐9, myocarditis

## Abstract

Adiponectin (APN) is a multifunctional adipocytokine that inhibits myocardial fibrosis, dilatation, and left ventricular (LV) dysfunction after myocardial infarction (MI). Coxsackievirus B3 (CVB3) myocarditis is associated with intense extracellular matrix (ECM) remodeling which might progress to dilated cardiomyopathy. Here, we investigated in experimental CVB3 myocarditis whether APN inhibits adverse ECM remodeling following cardiac injury by affecting matrix metalloproteinase (MMP) expression. Cardiac injury was induced by CVB3 infection in APN knockout (APN‐KO) and wild‐type (WT) mice. Expression and activity of MMPs was quantified by qRT‐PCR and zymography, respectively. Activation of protein kinases was assessed by immunoblot. In cardiac myocytes and fibroblasts APN up‐regulates MMP‐9 expression via activation of 5′ adenosine monophosphate‐activated protein kinase (AMPK) and extracellular signal‐regulated kinase (ERK)1/2 which function as master regulators of inflammation‐induced MMP‐9 expression. Correspondingly, APN further increased up‐regulation of MMP‐9 expression triggered by tumor necrosis factor (TNF)α, lipopolysaccharide (LPS) and R‐848 in cardiac fibroblasts. In vivo, compared to WT mice cardiac MMP‐9 activity and serum levels of carboxy‐terminal telopeptide of type I collagen (ICTP) were attenuated in APN‐KO mice in subacute (day 7 p.i.) CVB3 myocarditis. Moreover, on day 3 and day 7 post CVB3 infection splenic MMP‐9 expression was diminished in APN‐KO mice correlating with attenuated myocardial immune cell infiltration in subacute CVB3 myocarditis. These results indicate that APN attenuates adverse cardiac remodeling following cardiac injury by up‐regulating MMP‐9 expression in cardiac and immune cells. Thus, APN mediates intensified collagen cleavage that might explain inhibition of LV fibrosis and dysfunction.

## Introduction

Irreversible loss of cardiac myocytes caused by exogenous or endogenous pathogenic factors, hyperthrophy of the remaining myocytes and massive inflammatory infiltration of immune cells all contribute to changes of the myocardial extracellular matrix (ECM) associated with adverse remodeling of the left ventricular (LV) wall.

Since cardiac myocyte regeneration is insufficient in the adult heart (Oyama et al. 2013), damaged myocardium is replaced by parenchymal scar tissue (Kania et al. 2009). However, triggered by persistent inflammation this initially adaptive healing process turns into a maladaptive response resulting in cardiac fibrosis, LV hypertrophy/dilatation and stiffening (Schultheiss et al. 2011). The progress of adverse cardiac ECM remodeling essentially depends on cytokines released by resident cardiac cells as well as heart‐infiltrating immune cells (i.e., dendritic cells (DCs), monocytes, and T cells) that induce profibrotic changes in the expression pattern of ECM components and matrix metalloproteinases (MMPs) within the heart (D'Armiento 2002). Thus, adverse cardiac ECM remodeling associated with cardiac disease states such as myocardial infarction (MI), overload‐induced LV hyperthrophy and cardiomyopathy eventually results in LV dysfunction and manifestation of heart failure (Spinale 2007).

Cardiac fibrosis is a major indicator of adverse cardiac ECM remodeling and is characterized by excess collagen accumulation within the heart (Spinale 2007). The ECM turnover is tightly controlled by the coordinated degradation and synthesis of ECM components. Proteolytic enzymes belonging to the family of MMPs process ECM degradation via cleavage of collagens. In the heart eight types of MMPs (MMP‐1, MMP‐2, MMP‐3, MMP‐7, MMP‐8, MMP‐9, MMP‐13, and MMP‐14) are expressed (Spinale 2007) and cardiac fibroblasts constitute the key regulators of myocardial ECM structure, because they are the main producers of ECM components and MMPs. Results from clinical studies (Spinale et al. 2000) (Spinale et al. 1998) (Morita et al. 2006) and animal models (Rastogi et al. 2005) (Yarbrough and Spinale 2003) implicate MMP‐2, MMP‐3, MMP‐9, and MMP‐13 in profibrotic ECM remodeling associated with manifestation of heart failure.

Adiponectin (APN) is an abundant plasma cytokine, that is primarily expressed in adipocytes (Tilg and Moschen 2006). Moreover, APN expression has also been shown for endothelial cells, skeletal myocytes and cardiac cells (Tilg and Moschen 2006). APN exists as full length protein in several oligomeric forms or as proteolytic cleavage fragment in high concentrations of 3–30 *μ*g/mL in human plasma (Tilg and Moschen 2006). APN functions as important modulator of immune reactions exerting a wide spectrum of anti‐inflammatory effects (Tilg and Moschen 2006). Moreover, previous results from animal studies examining different cardiac disease states such as MI and hypertension/pressure overload have shown that APN attenuates adverse cardiac ECM remodeling (Shibata et al. 2004; Fujita et al. 2008; Shibata et al. 2007). Compared to wild‐type (WT) mice, APN knockout (APN‐KO) mice were characterized by exaggerated cardiac fibrosis and hyperthrophy, deteriorated LV function and increased mortality (Shibata et al. 2004; Fujita et al. 2008; Shibata et al. 2007). Moreover, in patients with virus‐negative DCMi high systemic APN levels are associated with improved outcome of LV end‐diastolic diameter and LV ejection fraction as indicators of attenuated cardiac dilatation (Bobbert et al. 2011).

Mechanistically, APN attenuates cardiac fibrosis via 5′ adenosine monophosphate‐activated protein kinase (AMPK)‐ and peroxisome proliferator‐activated receptor (PPAR)α‐mediated inhibition of collagen expression in cardiac fibroblasts (Fujita et al. 2008). Interestingly, severe hypertension‐induced LV hypertrophy in APN‐KO mice is associated with a reduced cardiac MMP‐2 expression compared to WT mice (Sam et al. 2010) indicating that APN might also inhibit profibrotic ECM remodeling by up‐regulating expression of collagen‐cleaving MMPs.

Here, we investigate whether APN inhibits adverse cardiac ECM remodeling following cardiac injury by affecting MMP expression employing the murine model of coxsackievirus B3 (CVB3) myocarditis. We show that APN up‐regulates MMP‐9 expression in cardiac fibroblasts and myocytes as well as infiltrating immune cells thus contributing to increased cardiac MMP‐9 activity in CVB3 myocarditis. Thus, increased cleavage of accumulating collagens associated with up‐regulation of MMP‐9 expression by APN might explain previously observed inhibitory effects of APN on adverse profibrotic ECM remodeling following cardiac injury.

## Material and Methods

### Animals and experimental model of CVB3 myocarditis

The initial breeding pairs of APN‐KO mice with a C57BL/6J background and corresponding C57BL/6J WT mice were purchased from Jackson Laboratories. The investigation was approved by the institutional ethics committee and the respective authorities in Berlin (Germany), it conformed to the NIH *Guide for the Care and Use of Laboratory Animals* (eighth edition, published 2011) and has been performed in accordance with the *Declaration of Helsink*i (seventh revision, published 2013). Animals were housed in cages with same sex littermates in climate‐controlled facilities (22°C, 40–50% relative humidity) of Forschungseinrichtung für Experimentelle Medizin (Berlin, Germany) on a 12 h light‐dark cycle with ad libitum food and water access. Breeding pairs were housed together until signs of pregnancy were noted, at which time the male was removed. Mice were euthanized by cervical dislocation. Before euthanization animals were anesthetized by inhalation of 2.0 vol% isoflurane (Abbot) for 5 min using an automatic delivery system (Rockmart). Adequate anesthesia was tested by monitoring withdrawal response to foot pinch.

For induction of experimental CVB3 myocarditis 6–8 weeks old male mice were infected by intraperitoneal injection of 200 *μ*L phosphate‐buffered saline (PBS) containing 5 × 10^5^ plaque forming units (PFU) of transfection‐derived purified CVB3 (Nancy strain; ATCC Number: VR‐30) (Jenke et al. 2014). Seven days p.i. infected APN‐KO and WT mice were compared with PBS‐treated mice of both groups. A minimum CVB3 positive strand copy number of 1 × 10^3^ per ng cDNA in left ventricular tissue specimens as determined by qRT‐PCR was used as threshold for inclusion of infected animals into the study. The CVB3 positive strand copy number was measured for all CVB3‐infected animals using triplicates of left ventricular cDNA samples (Jenke et al. 2014).

### Genotyping

Mouse pups were tail clipped for genotyping at an age of 5–8 days. Genomic DNA was extracted using DNeasy tissue kit (Qiagen) from isolated tail segments. WT and APN‐KO mice were then genotyped by PCR as described (Guo et al. 1832) using the following primers TGG ATG CTG CCA TGT TCC CAT (WT forward), CTT GTG TCT GTG TCT AGG CCT T (WT reverse), and CTC CAG ACT GCC TTG GGA (mutant reverse). Moreover, to confirm genotyping results for animals included in the study cardiac mRNA expression was measured by qRT‐PCR (cp. Section 3.6) using the TaqMan gene expression assay Mm00456425_m1 (Applied Biosystems) and APN plasma levels were quantified using a specific ELISA kit (R&D Systems).

### Cell culture and reagents

Neonatal cardiac fibroblasts and myocytes were prepared from hearts of 1–3 day old Wistar Unilever rats (randomly chosen male and female neonates, euthanization by decapitation) (Vetter et al. 1998). Recombinant human full‐length APN produced in a mammalian expression system was purchased from R&D Systems; measured endotoxin contamination (Kinetic‐QCL, Lonza) was below 10 pg/*μ*g protein. TLR ligands LPS and R‐848 were purchased from Enzo Life Sciences, recombinant rat TNFα from R&D Systems and enzyme inhibitors from Sigma‐Aldrich.

### Zymography

Gelatinolytic activities of MMP‐2 and MMP‐9 were determined in conditioned cell culture media and LV tissue lysates via zymograhy. Tissue lysates were generated using ready to use Cell Lysis Buffer (Cell Signaling). A quantity of 30 *μ*g of cardiac tissue protein or 20 *μ*L of conditioned media from cardiac myocytes and fibroblasts was mixed with sampling buffer (0.5 mol/L Tris‐HCl pH 6.8, 50% glycerol, 10% SDS, and 0.1% bromphenol blue). SDS‐PAGE was performed by running 10% polyacrylamide gels containing 0.1% gelatin at 150 V. SDS was removed from gels by incubation in renaturation solution containing 2.5% (v/v) Triton X‐100 for 60 min and gelatinolytic MMP activities were induced by incubation of gels in developing buffer (0.01 mol/L Tris‐base, 0.4 mol/L Tris‐HCl, 2 mol/L NaCl, 0.07 mol/L CaCl_2_, 0.1 mmol/L MgCl_2_, and 0.6% Brij‐35) at 37°C overnight. Gels were stained for 2 h with 0.5% (w/v) coomassie G250 and discolored for 30 min in 7% (v/v) acetic acid and 35% (v/v) methanol. Areas of gelatinolytic MMP activities were detected as clear bands against blue background. Band intensities were analyzed using ImageJ software (National Institutes of Health).

### Immunoblot

Protein from cardiac fibroblasts was extracted using Cell Lysis Buffer (Cell Signaling) supplemented with proteinase and phosphatase inhibitors (Complete Mini and PhosSTOP, Roche Applied Science). A quantity of 30 *μ*g protein was mixed with sampling buffer (375 mmol/L Tris‐HCl pH 6.8, 60% glycerol, 12.6% SDS, 600 mmol/L DTT, and 0.1% bromphenol blue) and boiled at 95°C for 5 min. SDS‐PAGE was performed by running 10% polyacrylamide gels at 200 V. Separated proteins were transferred to nitrocellulose membranes (GE Healthcare) via tank blot at 100 V for 90 min. The activation status of AMPK and ERK1/2 was quantified using monoclonal antibodies against phosphorylated AMPK (#2535) and ERK1/2 (#4370), total AMPK (#5831), and ERK1/2 (#4695) as well as corresponding horseradish peroxidase‐conjugated secondary antibodies (all Cell Signaling Technologies). Protein bands were visualized using a chemoluminescence system (Thermo Scientific). Band intensities were quantified using ImageJ software (National Institutes of Health).

### Quantitative real‐time polymerase chain reaction (qRT‐PCR)

RNA from tissues and cells was extracted using TRIzol (Invitrogen) and the RNeasy Mini Kit (Qiagen). RNA integrity was checked by 2100 Bioanalyzer (Agilent Technologies). qRT‐PCR was performed using High capacity cDNA Reverse Transcription Kit, TaqMan Universal PCR Master Mix, and TaqMan gene expression assays (*Rattus norvegicus*: MMP‐2 (Rn01538170_m1), MMP‐3 (Rn00591740_m1), MMP‐9 (Rn00579162_m1), MMP‐13 (Rn01448194_m1), GAPDH (Rn99999916_s1); *Mus musculus*: MMP‐9 (Mm00442991_m1), GAPDH (Mm99999915_g1), NKp46 (Mm01337324_g1), F4/80 (Mm00802529_m1), CD3z (Mm00446171_m1), CD4 (Mm00442754_m1), CD8a (Mm01182107_g1), HPRT1 (Mm01545399_m1) from Applied Biosystems.

### Inflammatory score and CD11b immunohistochemistry

Tissue Tec‐embedded frozen left ventricular tissue sections were used for histological measurements in LV tissue. Inflammatory infiltrates were quantified in H&E‐stained section high power fields (200x). Four sections were scored per animal. The inflammatory score was calculated by multiplication of the share of section area marked by immune cell infiltrates with the average degree of inflammatory foci. The degree of inflammatory foci was discriminated as follows: 0 = no immune cell infiltrates; 1= small inflammatory foci with few cells; 2 = medium grade foci with ≤100 immune cells; and 3 = severe inflammatory foci with ≥100 immune cells foci. CD11b immunohistochemistry was performed using a primary antibody for CD11b followed by the Envision horseradish peroxidase technique (DAKO). Results were quantified by digital image analyses of four sections per animal.

### Collagen type I degradation

Collagen type I degradation was determined via measurement of serum carboxy‐terminal telopepide type I collagen (ICTP) concentrations. For measurements, mouse serum was diluted 1:10 in PBS and ICTP abundance was measured employing a Mouse ICTP ELISA Kit (Abbexa, Cambridge, GB) according to the instructions of the manufacturer.

### Statistical analysis

**Figure 1:**
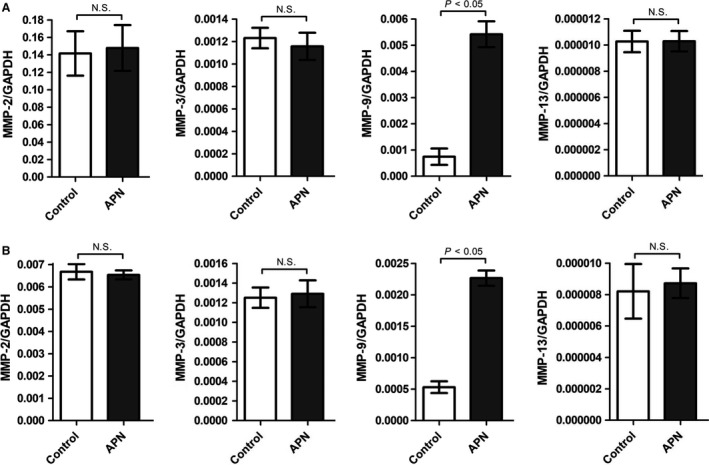
APN induces up‐regulation of MMP‐9 mRNA expression in cardiac fibroblasts and myocytes. (A) Cardiac fibroblasts were incubated with APN (20 *μ*g/mL) for 24 h. mRNA expression of MMP‐2, MMP‐3, MMP‐9, and MMP‐13 was measured by RT‐PCR. Results are presented as Mean ± SEM (*n* = 4 independent experiments). Statistical differences were assessed via Mann–Whitney *U* test. (B) Cardiac myocytes were incubated with APN (20 *μ*g/mL) for 24 h. mRNA expression of MMP‐2, MMP‐3, MMP‐9, and MMP‐13 was measured by RT‐PCR. Results are presented as Mean ± SEM (*n* = 4 independent experiments). Statistical differences were assessed via Mann–Whitney *U* test.

**Figure 2:**
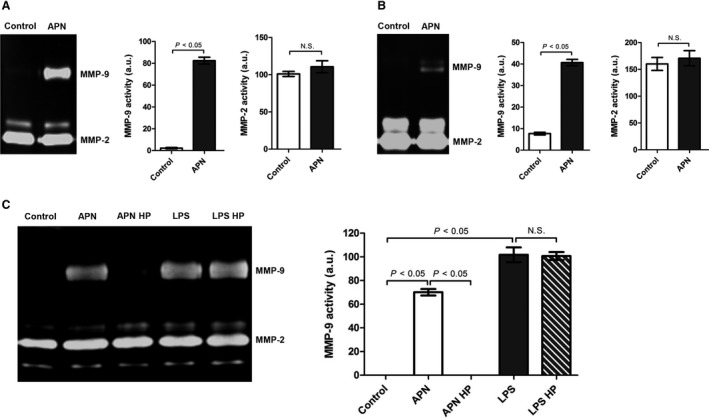
APN induces up‐regulation of MMP‐9 protein expression in cardiac fibroblasts and myocytes. Cardiac (A) fibroblasts and (B) myocytes were incubated with APN (20 *μ*g/mL) for 24 h. MMP activities in conditioned media were measured by zymography. Results are presented as Mean ± SEM in arbitrary units (*n* = 3 independent experiments). Statistical differences were assessed via Mann–Whitney *U* test. (C) Cardiac fibroblasts were incubated with APN (20 *μ*g/mL), heat processed APN (20 *μ*g/mL), LPS (1 *μ*g/mL), and heat processed LPS (1 *μ*g/mL) for 24 h. Heat processing (HP) was done at 95°C for 15 min. MMP activities in conditioned media were measured by zymography. Results are presented as Mean ± SEM in arbitrary units (*n* = 3 independent experiments). Statistical differences were assessed using the Kruskal–Wallis test followed by post hoc testing via Mann–Whitney *U* test for pair‐wise comparisons between individual groups.

GraphPad PRISM Version 5.04 was used for statistical data analysis. Results are expressed as mean ± SEM. Normality distribution of data was not assumed and in consequence nonparametric test were performed. For Figures 1A, B, and 2A, B statistical differences were assessed using Mann–Whitney U test. For all other figures statistical differences were assessed using the Kruskal–Wallis test followed by post hoc testing via Mann–Whitney *U* test for pair‐wise comparisons between individual groups. Differences were considered statistically significant at a one‐sided value of *P* < 0.05. Bonferroni adjustment was not performed because of the explorative nature of the study.

## Results

### APN induces up‐regulation of MMP‐9 expression in cardiac fibroblasts and myocytes via activation of AMPK and ERK1/2

Cardiac fibroblasts are major regulators of cardiac ECM remodeling because they produce a wide variety of ECM proteins including different types of MMPs (Spinale et al. 2000). To investigate whether APN inhibits adverse cardiac remodeling by modulating MMP activities within the heart, cardiac fibroblasts and myocytes were incubated with APN and expression levels of MMPs known to control profibrotic changes of LV structure associated with development of heart failure (Spinale et al. 2000; Spinale et al. 1998; Morita et al. 2006; Rastogi et al. 2005; Yarbrough and Spinale 2003) were analyzed by qRT‐PCR. Whereas APN had no influence on mRNA expression of MMP‐2, MMP‐3, and MMP‐13, it induced a significant up‐regulation of MMP‐9 mRNA expression in cardiac fibroblasts (Fig. 1A) and myocytes (Fig. 1B). Moreover, the zymographic analysis of conditioned media from cultured cardiac fibroblasts (Fig. 2A) and myocytes (Fig. 2B) revealed a substantial increase in gelatinolytic MMP‐9 activity (molecular weight: 95 kDa) after incubation with APN, indicating that APN up‐regulates MMP‐9 protein expression and subsequent enzyme secretion in both cell types.

Lipopolysaccharide (LPS) is a potent inducer of MMP‐9 expression and represents a major source for contaminations in recombinant proteins. To ensure that the observed up‐regulation of MMP‐9 expression following APN incubation is not due to endotoxin contamination, cardiac fibroblasts were incubated with untreated as well as heat processed (95°C for 15 min) APN and LPS before MMP activities were determined. However, heat processing completely abolished the APN‐induced up‐regulation of MMP‐9 expression in cardiac fibroblasts, it had no influence on the up‐regulation triggered by LPS (Fig. 2C), indicating that APN itself induces up‐regulation of MMP‐9 expression.

**Figure 3:**
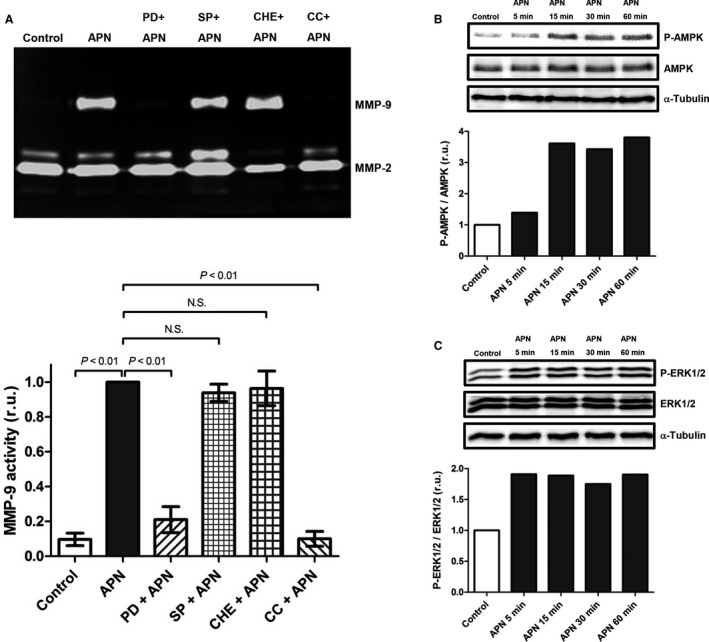
APN induces up‐regulation of MMP‐9 protein expression in cardiac fibroblasts via activation of AMPK and ERK1/2. (A) Cardiac fibroblasts were pretreated with ERK1/2 inhibitor PD 098059 (PD, 20 *μ*mol/L), JNK inhibitor SP 600125 (SP, 20 *μ*mol/L), PKC inhibitor Cherylerythrine (Che, 5 *μ*mol/L), and AMPK inhibitor Compound C (CC, 20 *μ*mol/L) for 1 h followed by stimulation with APN (20 *μ*g/mL) for 24 h. Upper panel: MMP activities in conditioned media were measured by gelatin zymography. Lower panel: Bar graph indicating quantified gelantinolytic MMP‐9 activities. Results are presented as Mean ± SEM in relative units (*n* = 6 independent experiments). Statistical differences were assessed using the Kruskal–Wallis test followed by post hoc testing via Mann–Whitney U test for pair‐wise comparisons between individual groups. (B) Cardiac fibroblasts were incubated with APN (20 *μ*g/mL) for time periods of 5–60 min. Phosphorylation status of AMPK was determined by immunoblot. Quantification of α‐Tubulin expression was used as loading control. (C) Cardiac fibroblasts were incubated with APN (20 *μ*g/mL) for time periods of 5–60 min. Phosphorylation status of ERK1/2 was determined by immunoblot. Quantification of α‐Tubulin expression was used as loading control.

AMPK, c‐Jun NH_2_‐terminal kinase (JNK), protein kinase C (PKC), and extracellular signal‐regulated kinase (ERK)1/2 all have been shown to act as signaling mediators for up‐regulation of MMP‐9 expression (Xie et al. 2004; Suzuki et al. 2004). To study a potential involvement of AMPK, JNK, PKC, and ERK1/2 signaling in the APN‐induced up‐regulation of MMP‐9 expression, cardiac fibroblasts were pretreated with Compound C (AMPK inhibitor), SP 600125 (JNK inhibitor), Cherylerythrine (PKC inhibitor), and PD 098059 (ERK1/2 inhibitor). The APN‐induced up‐regulation of MMP‐9 expression in cardiac fibroblasts was abolished by PD 098059 and Compound C (Fig. 3A), while pretreatment with SP600125 and Cherylerythrine had no effect. Accordingly, incubation of cardiac fibroblasts with APN for periods from 15 to 60 min resulted in substantial phosphorylation/activation of both AMPK (Fig. 3B) and ERK1/2 (Fig. 3C).

### AMPK and ERK1/2 are master regulators of inflammation‐induced up‐regulation of MMP‐9 expression in cardiac fibroblasts

**Figure 4:**
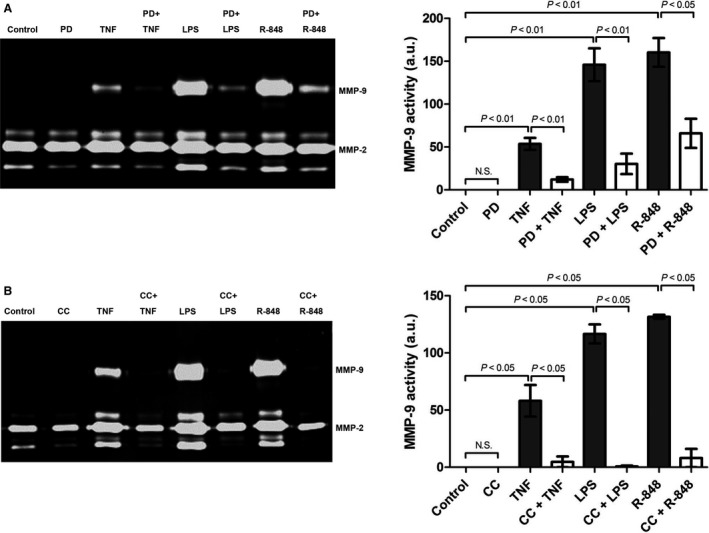
AMPK and ERK1/2 act as master regulators of inflammation‐induced up‐regulation of MMP‐9 expression in cardiac fibroblasts. Cardiac fibroblasts were pre‐treated with (A) ERK1/2 inhibitor PD 098059 (PD, 20 *μ*mol/L) or (B) AMPK inhibitor Compound C (CC, 20 *μ*mol/L) for 1 h followed by stimulation with TNFα (10 ng/mL), LPS (1 *μ*g/mL) or R‐848 (5 *μ*g/mL) for 24 h. Left panel: MMP activities in conditioned media were measured by gelatin zymography. Right panel: Bar graph indicating quantified gelantinolytic MMP‐9 activities. Results are presented as Mean ± SEM in arbitrary units (PD: *n* = 5 independent experiments, CC: *n* = 4 independent experiments). Statistical differences were assessed using the Kruskal–Wallis test followed by post hoc testing via Mann–Whitney *U* test for pair‐wise comparisons between individual groups.

Cardiac expression of MMP‐9 is up‐regulated by proinflammatory stimuli such as cytokines, reactive oxygen species (ROS) and Toll‐like receptor (TLR) ligands that typically accumulate at sites of cardiac injury (Siwik et al. 2000). To examine whether AMPK and ERK1/2 control the up‐regulation of MMP‐9 expression by proinflammatory stimuli, cardiac fibroblasts were pretreated with AMPK inhibitor Compound C and ERK1/2 inhibitor PD 098059. Up‐regulation of MMP‐9 expression in cardiac fibroblasts following incubation with the cytokine tumor necrosis factor (TNF)α and the TLR ligands LPS and R‐848 was significantly attenuated by PD 098059 (Fig. 4A) and completely abolished by Compound C (Fig. 4B). Thus, AMPK and ERK1/2 function as master regulators of inflammation‐induced up‐regulation of MMP‐9 expression in cardiac fibroblasts.

### APN increases up‐regulation of MMP‐9 expression in cardiac fibroblasts in response to proinflammatory stimuli

**Figure 5:**
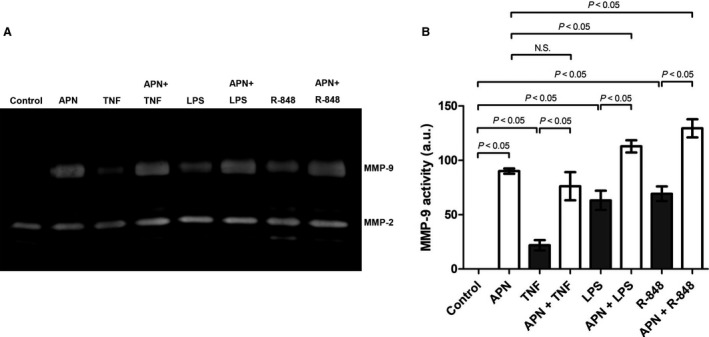
APN enhances up‐regulation of MMP‐9 expression in cardiac fibroblasts in response to proinflammatory stimulation. Cardiac fibroblasts were pretreated with APN (20 *μ*g/mL) for 6 h followed by stimulation with TNFα (10 ng/mL), LPS (1 *μ*g/mL), and R‐848 (5 *μ*g/mL) for 18 h. Left panel: MMP activities in conditioned media were measured by gelatin zymography. Right panel: Bar graph indicating quantified gelantinolytic MMP‐9 activities. Results are presented as Mean ± SEM in arbitrary units (*n* = 3 independent experiments). Statistical differences were assessed using the Kruskal–Wallis test followed by post hoc testing via Mann–Whitney *U* test for pair‐wise comparisons between individual groups.

To examine whether APN also modulates the up‐regulation of MMP‐9 expression in response to proinflammatory stimuli cardiac fibroblasts were incubated with TNFα, LPS, and R‐848 in the presence or absence of APN. APN preincubation significantly enhanced the up‐regulation of MMP‐9 expression in cardiac fibroblasts in response to TLR agonists, such as LPS and R‐848 (Fig. 5). These data indicate that APN up‐regulates MMP‐9 expression in cardiac fibroblasts under baseline and proinflammatory conditions.

### APN‐KO mice exhibit an attenuated up‐regulation of cardiac and splenic MMP‐9 expression accompanied by alleviated cardiac inflammation in CVB3 myocarditis

**Figure 6:**
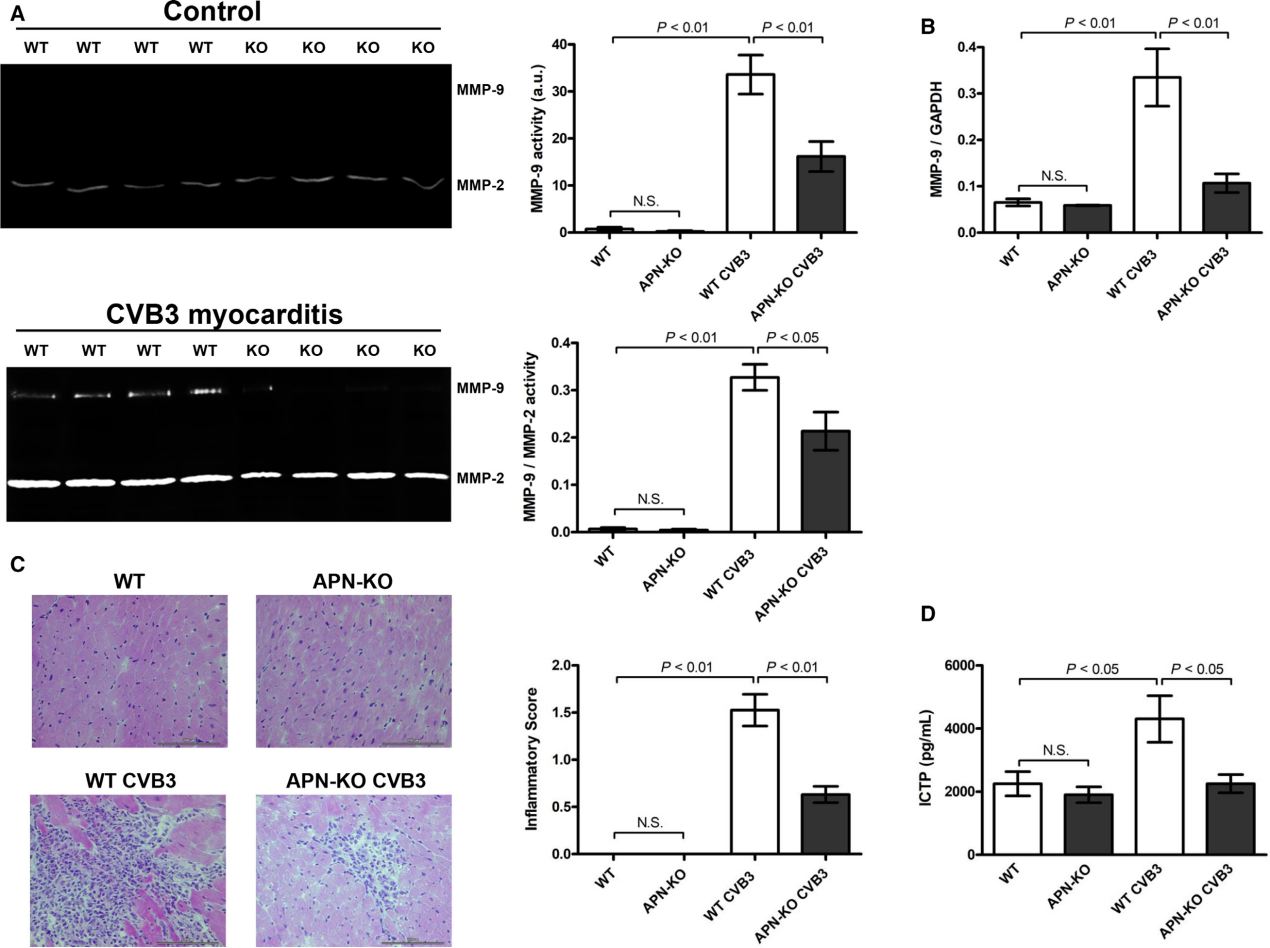
APN‐KO mice exhibit an attenuated up‐regulation of cardiac and splenic MMP‐9 expression accompanied by alleviated cardiac inflammation in CVB3 myocarditis. (A) MMP‐9 and MMP‐2 activities in cardiac tissue of CVB3‐infected WT and APN‐KO mice on day 7 p.i. were measured by zymography. Areas of gelatinolytic MMP‐9 and MMP‐2 activities are visible in zymography gels as clear bands against blue background. Top right panel: Bar graph indicating quantified gelantinolytic MMP‐9 activities. Down right panel: Bar graph indicating the ratio of quantified gelantinolytic MMP‐9 and MMP‐2 activities. Results are presented as Mean ± SEM in arbitrary units (*n* = 8–11 animals per group). Statistical differences were assessed using the Kruskal–Wallis test followed by post hoc testing via Mann–Whitney *U* test for pair‐wise comparisons between individual groups. (B) Splenic MMP‐9 expression of CVB3‐infected WT and APN‐KO mice on day 7 p.i. was measured by qRT‐PCR. Results are presented as Mean ± SEM (*n* = 5–6 animals per group). Statistical differences were assessed using the Kruskal–Wallis test followed by post hoc testing via Mann–Whitney *U* test for pair‐wise comparisons between individual groups. (C) Inflammatory cell infiltration in hearts of WT and APN‐KO mice on day 7 p.i. was scored in H & E‐stained LV tissue sections. Results are presented as Mean ± SEM (*n* = 10–14 animals per group). Statistical differences were assessed using the Kruskal–Wallis test followed by post hoc testing via Mann–Whitney *U* test for pair‐wise comparisons between individual groups. (D) Concentrations of Carboxy‐terminal telopepide type I collagen (ICTP) in sera of CVB3‐infected WT and APN‐KO mice on day 7 p.i. were quantified by ELISA. Results are presented as Mean ± SEM (*n* = 8–11 animals per group). Statistical differences were assessed using the Kruskal–Wallis test followed by post hoc testing via Mann–Whitney *U* test for pair‐wise comparisons between individual groups.

Experimental CVB3 myocarditis is an established model of inflammatory heart disease, characterized by extensive cardiac ECM remodeling (Marchant and McManus 2009). In order to examine the effects of APN on cardiac MMP‐9 expression in inflammatory heart disease and to corroborate our in vitro results, WT and APN‐KO mice were subjected to CVB3 infection inducing viral myocarditis. In hearts of uninfected control mice only marginal MMP‐9 expression was detected that did not differ between WT or APN deficient mice (Fig. 6A) in accordance with a compensated phenotype of APN‐KO mice under basal conditions (Shibata et al. 2004). In subacute CVB3 myocarditis on day 7 p.i. cardiac MMP‐9 activity as well as MMP‐9/MMP‐2 activity ratio were significantly up‐regulated in both WT and APN‐KO mice compared to baseline levels. However, the infection‐induced up‐regulation of MMP‐9 activity and MMP‐9/MMP‐2 actvity ratio was significantly attenuated in hearts of APN‐KO mice, indicating that systemic APN deficiency attenuates up‐regulation of cardiac MMP‐9 expression in inflammatory heart disease (Fig. 6A, respectively). These data corroborate our in vitro findings in cardiac fibroblasts and myocytes.

**Figure 7:**
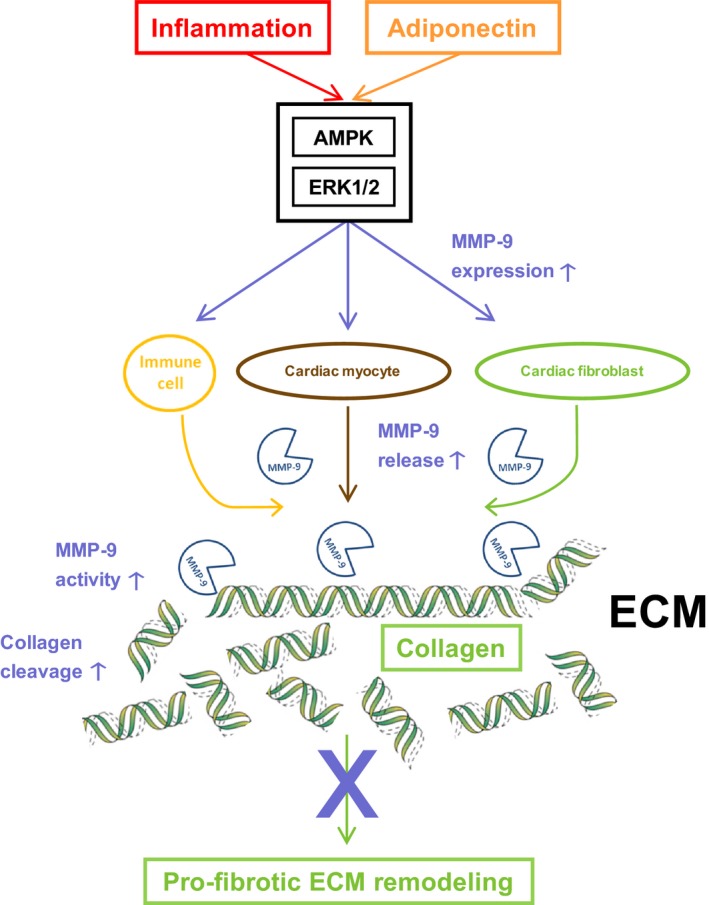
APN attenuates profibrotic ECM remodeling following cardiac injury by up‐regulating MMP‐9 expression. Following cardiac injury APN‐induced up‐regulation of MMP‐9 expression in cardiac fibroblasts and myocytes as well as heart‐infiltrating immune cells triggers increased cleavage of accumulating collagens and augmented ECM turnover. Thus, APN attenuates development of cardiac fibrosis resulting in LV dilatation and dysfunction.

Besides cardiac fibroblasts infiltrating immune cells, for example,. DCs and T cells ‐ represent major sources of MMP expression in inflammatory heart disease (St‐Pierre and Potworowski 2000). At baseline there was no difference in splenic MMP‐9 mRNA expression of uninfected WT and APN‐KO mice. (Fig. 6B). In acute (day 3 p.i., data not shown) and subacute (day 7 p.i.) CVB3 myocarditis splenic MMP‐9 expression in WT mice was significantly up‐regulated compared to baseline levels (Fig. 6B). However, this effect was significantly attenuated in APN‐KO mice, respectively. Moreover, attenuated MMP‐9 expression in immune cells of CVB3‐infected APN‐KO mice was associated with a diminished myocardial immune cell infiltration on day 7 p.i. compared to infected WT mice as indicated by H&E staining of LV tissue sections (Fig. 6C), mRNA levels of various immune cell‐specific surface markers such as NKp46, F4‐80, CD3z, CD4, CD8a and infiltration of CD11b+ immune cells in LV tissue (Table 1, respectively). MMP‐9‐mediated cleavage of collagen type I results in release of carboxy‐terminal telopepide type I collagen (ICTP) as degradation product (Garnero et al. 2003). Correspondingly, the attenuated up‐regulation of cardiac MMP‐9 expression in APN‐KO mice subjected to CVB3 myocarditis was accompanied by significantly diminished ICTP serum levels compared to CVB3‐infected WT mice (Fig. 6D). Taken together, diminished MMP‐9 release from both resident cardiac and infiltrated immune cells seems to contribute to attenuated up‐regulation of cardiac MMP‐9 expression as well as decreased collagen type I cleavage in CVB3‐infected APN‐KO mice compared to their WT littermates (Fig. 7).

**Table 1:** APN‐KO mice exhibit attenuated myocardial immune cell infiltration in CVB3 myocarditis

Method	Measure	WT	APN‐KO	WT CVB3	APN‐KO CVB3
qRT‐PCR	Nkp46/HPRT1	0.0011 ± 0.0001	0.0013 ± 0.0001	0.0481 ± 0.0088^a^	0.0224 ± 0.0033^b^
qRT‐PCR	F4‐80/HPRT1	0.1984 ± 0.0133	0.2096 ± 0.0088	1.3049 ± 0.1679^a^	0.7939 ± 0.0520
qRT‐PCR	CD3Z/HPRT1	0.0031 ± 0.0002	0.0038 ± 0.0002	0.0630 ± 0.0135	0.0300 ± 0.0038^b^
qRT‐PCR	CD4/HPRT1	0.0040 ± 0.0002	0.0034 ± 0.0004	0.0451 ± 0.0102^a^	0.0206 ± 0.0027^b^
qRT‐PCR	CD8a/HPRT1	0.0015 ± 0.0003	0.0019 ± 0.0002	0.0460 ± 0.0082	0.0223 ± 0.0034^b^
IHC	CD11b+cells/mm^2^	11.82 ± 2.10	8.82 ± 2.26	813.73 ± 124.04^a^	297.65 ± 66.93

Mean ± SEM

a WT CVB3 versus WT

b APN‐KO CVB3 versus WT CVB3

## Discussion

Our study demonstrates for the first time that APN up‐regulates MMP‐9 expression in cardiac fibroblasts and myocytes as well as heart‐infiltrating immune cells thereby contributing to elevated cardiac MMP‐9 expression following local tissue injury associated with CVB3 myocarditis. Persistently enhanced cardiac MMP‐9 expression/activity in the presence of APN results in increased cleavage of accumulating collagens and augmented ECM turnover in CVB3 myocarditis. Increased collagen cleavage associated with up‐regulation of MMP‐9 expression might represent a novel mechanism of anti‐fibrotic action mediated by APN that could explain previously observed inhibitory effects on adverse ECM remodeling in inflammatory (Bobbert et al. 2011) as well as noninflammatory (Fujita et al. 2008; Shibata et al. 2007; Sam et al. 2010) heart disease. APN has recently been demonstrated to up‐regulate expression of MMP‐1, MMP‐3, and MMP‐13 in human chondrocytes (Kang et al. 2010; Tong et al. 2011) and synovial fibroblasts (Choi et al. 2009; Ehling et al. 2006). No effect of APN on expression levels of other MMPs was observed in our study. However, consistent to our results APN has been shown to specifically up‐regulate expression of MMP‐9 in hepatocytes (Wanninger et al. 2011) and microvascular endothelial cells (Adya et al. 2012) in vitro. Moreover, APN also up‐regulates MMP‐9 expression in murine chondrocytes (Lago et al. 2008). Thus, the APN‐induced regulation pattern of MMP expression seems to be cell type‐specific.

Here, we show for the first time an APN‐induced up‐regulation of MMP‐9 mRNA as well as protein expression in cardiac myocytes and fibroblasts. Moreover, APN‐induced up‐regulation of MMP‐9 expression is associated with an increased enzymatic MMP‐9 activity in cell culture supernatants for both cell types. This is in contrast to results of a recent in vitro study demonstrating an APN‐induced up‐regulation of MMP‐2 activity in culture supernatants of cardiac fibroblasts (Dadson et al. 2014). However, Dadson et al. (2014) showed that the observed up‐regulation of extracellular MMP‐2 activity is a short‐time effect resulting from an increased cell surface translocation of MMP‐14 which cleaves pro‐MMP‐2 to its active form and not as consequence of a modulation of MMP‐2 expression levels. Furthermore, the discordant findings of APN‐induced MMP regulation in cardiac fibroblasts and other cell types (Kang et al. 2010; Tong et al. 2011; Choi et al. 2009; Ehling et al. 2006) might be related to different isoform (globular vs. full‐length), sources (in‐house production vs. external sourcing) and expression systems (bacterial vs. mammalian) of recombinant APN used in these studies. In this context endotoxin contaminations are of special concern for recombinant APN because production of endotoxin‐free APN is very challenging (Turner et al. 2009; Peake et al. 2006). However, heat processing of recombinant APN in our control experiment provides clear evidence that the observed up‐regulation of MMP‐9 expression in cardiac fibroblasts is in fact induced by APN.

APN‐induced up‐regulation of MMP‐9 expression in cardiac fibroblasts and myocytes is mediated by protein kinases AMPK and ERK1/2 which have been recently shown to act as signaling mediators for the up‐regulation of MMP‐9 expression (Xie et al. 2004; Wang et al. 2012). Moreover, incubation of cardiac fibroblasts with APN resulted in substantial activation of AMPK and ERK1/2 via phosphorylation, which is consistent with previous studies identifying these kinases as central mediators of APN signaling (Tilg and Moschen 2006; Lee et al. 2008). In accordance with our results in cardiac fibroblasts, the APN‐induced up‐regulation of MMP‐9 expression in microvascular endothelial cells is also AMPK‐dependent (Adya et al. 2012). Moreover, AMPK has been shown to mediate the APN‐induced up‐regulation of MMP‐1, MMP‐3 and MMP‐13 expression in chondrocytes (Kang et al. 2010).

Following cardiac injury, local cytokines and endogenously released TLR‐ligands modulate cardiac fibroblast function by changing the expression pattern of ECM components and MMPs (Spinale et al. 2000). Particularly, the MMP‐9 expression in cardiac fibroblasts is up‐regulated in response to proinflammatory stimuli (Siwik et al. 2000). Using the proinflammatory cytokine TNFα as well as the TLR ligands LPS and R‐848 we identified AMPK and ERK1/2 as master regulators of inflammation‐induced up‐regulation of MMP‐9 expression in cardiac fibroblasts. Accordingly, APN which activates AMPK and ERK1/2 in cardiac fibroblasts further enhanced the observed inflammatory up‐regulation of MMP‐9 expression following stimulation by TLR agonists. Taken together, these in vitro results demonstrate that APN enhances the up‐regulation of MMP‐9 expression in cardiac fibroblasts in response to proinflammatory stimuli and injury signals.

Cardiac injury caused by pathogen‐induced myocarditis is characterized by compensatory remodeling of the myocardial ECM. Several studies have demonstrated an up‐regulation of cardiac MMP‐9 expression and activity in experimental CVB3 myocarditis (Marchant and McManus 2009). Proinflammatory cytokines released from heart infiltrating immune cells and resident cardiac cells as well as viral and endogenous TLR ligands trigger the up‐regulation of cardiac MMP‐9 expression (Marchant and McManus 2009). In the in vivo‐experiments of this study, cardiac ECM remodeling was compared in APN‐KO and WT mice in experimental CVB3 myocarditis. Corroborating our in vitro‐findings, cardiac MMP‐9 activity in subacute CVB3 myocarditis (i.e., on day 7 p.i.) was significantly attenuated in APN‐KO mice. This observation might in part result from a diminished MMP‐9 release by heart infiltrating immune cells (St‐Pierre and Potworowski 2000). In this regard, the up‐regulation of splenic MMP‐9 expression following CVB3 infection was significantly attenuated in APN‐deficient animals compared to WT mice. As MMP‐9 has been shown to facilitate trans‐endothelial immune cell migration into the heart (St‐Pierre and Potworowski 2000), diminished MMP‐9 release from activated immune cells in APN‐KO mice might attenuate their subsequent cardiac infiltration and accumulation alleviating inflammation in subacute CVB3 myocarditis. Taken together, reduced MMP‐9 expression in immune cells of APN‐KO mice might influence cardiac ECM remodeling in CVB3 myocarditis by two distinct mechanisms, that is, attenuation of collagen degradation and inhibition of immune cell‐mediated tissue injury. Correspondingly, Cheung et al. (2008) demonstrated a protective effect of MMP‐9 in CVB3‐induced myocarditis. In this study, MMP9‐KO mice showed enhanced myocardial injury, increased inflammatory infiltrates and increased fibrosis within the heart on day 9 postinfection associated with decreased cardiac output. Moreover, inhibition of tissue inhibitor of matrix metalloproteinases (TIMP)‐1, an inhibitor of MMP‐9, was associated with amelioration of CVB3 myocarditis (Crocker et al. 2007).

Thus, the in vitro and in vivo results of our study provide a novel mechanism how APN might inhibit fibrosis following cardiac injury. Previous results from animal studies examining different cardiac disease states such as MI and hypertension/pressure overload showed attenuation of adverse cardiac ECM remodeling by APN (Shibata et al. 2004; Fujita et al. 2008; Shibata et al. 2007). Compared to WT mice, APN‐KO mice were characterized by exaggerated cardiac fibrosis and hyperthrophy, deteriorated LV function and increased mortality in these experimental diseases. Until today, direct inhibition of collagen expression in cardiac fibroblasts mediated by APN via activation of AMPK‐ and PPARα was the only mechanism explaining the observed anti‐fibrotic action of APN (Fujita et al. 2008). In our study, the APN‐induced up‐regulation of cardiac MMP‐9 expression results in increased MMP‐9‐mediated collagen cleavage thus inhibiting adverse profibrotic remodeling of the cardiac ECM. This novel mechanism complements data from previous studies attributing the anti‐fibrotic action of APN to an inhibition of cardiac collagen expression (Fujita et al. 2008).

Our hypothesis is supported by other animal studies examining myocardial remodeling in hypertension/pressure overload‐induced LV hypertrophy which is typically associated with cardiac fibrosis (Weber and Brilla 1991). These studies reveal that APN‐KO mice exhibit an aggravated LV hypertrophy of concentric character which is characterized by increased wall thickness and cardiac fibrosis finally resulting in impaired LV systolic dysfunction and increased mortality (Shibata et al. 2004; Fujita et al. 2008; Shibata et al. 2007; Shimano et al. 2010; O'Shea et al. 2010) compared to WT mice. Moreover, the observed phenotypic differences were attenuated by reconstitution of APN‐KO mice via adenoviral gene transfer of APN (Shibata et al. 2004). Importantly, myocardial hyperthrophy of APN‐KO mice was associated with a significantly diminished phosphorylation of cardiac AMPK (Shimano et al. 2010) and a significantly reduced cardiac MMP‐9 expression compared to WT mice (O'Shea et al. 2010). Taken together, these results indicate that the APN‐induced and AMPK‐mediated up‐regulation of cardiac MMP‐9 expression prevents adverse profibrotic hypertrophic LV remodeling in response to hypertension/pressure overload. Correspondingly, thiazolidinedione‐induced up‐regulation of systemic APN levels in WT mice was shown to attenuate hypertension/pressure overload‐induced LV hyperthrophy and fibrosis (Li et al. 2010). Likewise, thiazolidinedione‐induced up‐regulation of systemic APN levels has been shown to attenuate development of murine experimental renal fibrosis (Fujiu and Nagai 2013).

There are several clinical studies demonstrating an equivalent association between APN, MMP‐9 and tissue fibrosis as observed in murine experimental disease models. A recent study examining obese children revealed significantly reduced APN plasma concentrations associated with decreased MMP‐9 plasma levels compared to nonobese controls (Belo et al. 2012) and pathological expansion of adipose tissue in obese individuals which is typically accompanied by a substantial reduction in the local APN expression (Milan et al. 2002) triggers adipose tissue fibrosis (Sun and Scherer 2010).

Patients suffering from diffuse cutaneous systemic sclerosis are characterized by a decreased MMP‐9 serum activity and diminished MMP‐9 expression of skin fibroblasts which is accompanied by intensified skin fibrosis compared to healthy controls (Lakota et al. 2012; Fuzii et al. 2008). Simultaneously, systemic APN levels in diffuse cutaneous systemic sclerosis patients have been demonstrated to correlate inversely with the extent of skin fibrosis (Lakota et al. 2012).

With respect to hepatic fibrosis similar associations were identified. In patients suffering from chronic hepatitis C virus infections serum levels of MMP‐9 are negatively correlated with histological severity of fibrotic tissue cirrhosis (Badra et al. 2010). Moreover, insulin resistance which is associated with reduced systemic APN levels (Lihn et al. 2005) correlates with increased severity of hepatic fibrosis in these patients (Moucari et al. 2008). Accordingly, lower APN serum levels are inversely associated with advanced hepatic fibrosis in nondiabetic (Musso et al. 2005) and type 2 diabetic (Leite et al. 2013) patients with nonalcoholic steatohepatitis.

Finally, a study examining patients with hypertension – a disease state typically associated with low systemic APN levels (Imatoh et al. 2008) and LV hyperthrophy (Weber and Brilla 1991) – revealed that thiazolidindione‐induced up‐regulation of APN plasma levels improves the LV diastolic function (Horio et al. 2005). Moreover, in patients with virus‐negative DCMi high systemic APN levels indicate a favorable outcome of cardiac dilatation as quantified by decreased LV diastolic diameter and increased LV ejection fraction (Bobbert et al. 2011).

In conclusion, by demonstrating that APN up‐regulates MMP‐9 expression in resident cardiac and infiltrating immune cells the results of our study provide a novel mechanism how APN might inhibit adverse cardiac remodeling associated with MI, myocarditis and overload states. Supplementary to a direct inhibition of local collagen expression mediated by APN (Fujita et al. 2008), the APN‐induced up‐regulation of cardiac MMP‐9 activity results in increased cleavage of accumulating collagens and augmented ECM turnover that contributes to attenuation of LV fibrosis and dysfunction following cardiac injury. Several other MMPs and their inhibitors contribute to ECM remodeling in vivo. Missing data of the complex interplay of MMPs and TIMPs is a limitation of our in vivo study. However, our in vitro results clearly show an APN‐induced upregulation of MMP9‐ in cardiac cells that is corraborated in the in vivo model of CVB3 myocarditis. Other limitations of the study are the effects of APN on glucose metabolism, adipogenesis and energy homeostasis that are absent in APN‐deficient mice. These metabolic effects might have contributed to the observed changes in remodeling.

## Conflict of Interest

On behalf of all authors, the corresponding author states that there is no conflict of interest.
